# Effects of dietary and physical activity interventions on generic and cancer-specific health-related quality of life, anxiety, and depression in colorectal cancer survivors: a randomized controlled trial

**DOI:** 10.1007/s11764-020-00864-0

**Published:** 2020-02-18

**Authors:** Mandy Ho, Judy W. C. Ho, Daniel Y. T. Fong, C. F. Lee, Duncan J. Macfarlane, Ester Cerin, Antoinette M. Lee, Sharron Leung, Wynnie Y. Y. Chan, Ivy P. F. Leung, Sharon H. S. Lam, Natural Chu, Aliki J. Taylor, Kar-Keung Cheng

**Affiliations:** 1 School of Nursing, The University of Hong Kong, 4/F William M.W. Mong Block, 21 Sassoon Road, Pok Fu Lam, Hong Kong; 2 Department of Surgery, The University of Hong Kong, Pok Fu Lam, Hong Kong; 3 Centre for Sports and Exercise, The University of Hong Kong, Pok Fu Lam, Hong Kong; 4 School of Public Health, The University of Hong Kong, Pok Fu Lam, Hong Kong; 5 Mary MacKillop Institute for Health Research, Australia Catholic University, Melbourne, Australia; 6 Department of Psychology, The University of Hong Kong, Pok Fu Lam, Hong Kong; 7 School of Nursing, Hong Kong Baptist Hospital, Kowloon Tong, Hong Kong; 8 School of Professional and Continuing Education, The University of Hong Kong, Pok Fu Lam, Hong Kong; 9 Department of Dietetics, Queen Elizabeth Hospital, Kowloon, Hong Kong; 10 Institute of Applied Health Research, University of Birmingham, Birmingham, UK

**Keywords:** Health-related quality of life, Anxiety, Depression, Colorectal cancer, Cancer survivorship, Dietary intervention, Physical activity intervention

## Abstract

**Purpose:**

To assess the effects of dietary and physical activity (PA) interventions on generic and cancer-specific quality of life (QoL), anxiety, and depression levels among adult Chinese colorectal cancer (CRC) survivors.

**Methods:**

Two-hundred twenty-three adult CRC survivors within 1 year of completion of primary cancer treatment were randomized to receive dietary, PA or combined intervention, or usual care for a 12 monthduration, under a 2 (diet vs usual care) × 2 (PA vs usual care) factorial design. Generic and cancer-specific QoL was assessed using a Chinese version 12-Item Short Form Health Survey (SF-12) and the Functional Assessment of Cancer Therapy-Colorectal (FACT-C) scale, respectively. Anxiety and depression was assessed using the Hospital Anxiety and Depression Scale at baseline, 6, 12, 18, and 24 months. Linear mixed models were used for examining the intervention effects.

**Results:**

Participants receiving dietary intervention experienced a significant improvement in the generic measure of QoL (SF-6D utility scores, mean difference 0.042, 95%CI 0.03 to 0.081) at 12 months, the cancer-specific QoL scores (mean difference 3.09, 95%CI 0.13 to 6.04), and levels of depression (*P* = 0.015) at both 12 and 24 months follow-up. Participants receiving PA intervention only demonstrated a significant improvement in SF-6D utility index (mean difference 0.039, 95%CI 0.002 to 0.077) and physical functioning (mean difference 2.85, 95%CI 1.00 to 4.70) at 6 months.

**Conclusions:**

Dietary intervention improved the generic and cancer-specific QoL and depression in CRC survivors.

**Trial registration:**

The study was prospectively registered on 17 October 2012 at ClinicalTrials.gov (NCT01708824).

**Implications for Cancer Survivors:**

CRC survivors can benefit from dietary interventions in alleviating depression and improving overall health-related QoL.

## Introduction

Colorectal cancer (CRC) is a leading burden of disease worldwide. CRC was the third most common cancer in men and the second most common cancer in women with over 1.8 million new cases, accounting for 10.1% of total cancer burdens, in 2018 [1] . The burden of CRC is expected to increase by 60% with more than 2.2 million new cases by 2030 [2]. CRC is the most common cancer in Hong Kong with 5635 new cases in 2017. The crude incidence rate of CRC in Hong Kong increased from 48.2 per 100,000 in 2000 to 76.2 per 100,000 in 2017 [3].

CRC and its treatment often produce significant impacts on quality of life (QoL) and mental well-being in CRC survivors. In general, cancer survivors report greater mental health needs, higher levels of anxiety and depression, and poor physical and mental health-related QoL [4, 5]. A population-based survey conducted in the USA reported that on average, the health-related QoL score of cancer survivors was more than 1 standard deviation below the population mean [6]. With advances in cancer detection and treatment, CRC survivors are now living longer. This underscores the need for finding effective ways to improve both the short-term and long-term health-related QoL and mental health of CRC survivors.

Evidence from observational studies indicates that diet and physical activity (PA) are closely linked to health-related QoL in several types of cancer survivors including CRC [7, 8]. Evidence is emerging that cancer survivors with a greater adherence to lifestyle behavior recommendations report improved levels of health-related QoL [7]. These findings point to the potential positive impact of PA and healthy diet on the health-related QoL in this population. A few studies have investigated the effects of lifestyle intervention (diet plus PA interventions, with intervention duration ranging from 6 weeks to 6 months) on the health-related QoL and mental well-being in CRC survivors [9–11]. A recent systematic review including five randomized controlled trials published between 2003 and 2014 demonstrated the absence of a significant effect of PA intervention on QoL outcomes, with the exception of one trial reporting an association with improved physical well-being [12]. All of these studies were conducted in Caucasian populations with intervention duration up to 6 months. It is not known whether the findings from these trials can be generalized to the Asian population. Also, the effects of a longer-term PA intervention were uncertain. This type of information is especially important due to the rising trend of CRC in Asian countries.

Dietary factors play an important part in CRC recovery following primary treatment [13]. However, to our knowledge, no study has explored the role of dietary intervention on both generic and cancer-specific QoL and levels of anxiety and depression in CRC survivors. The “Moving Bright, Eating Smart” study was the first multicenter RCT to assess the efficacy of dietary and PA interventions targeted at dietary and PA behaviors among adult Chinese CRC survivors [14]. This paper reports the effects of dietary and PA interventions on generic health-related QoL, CRC-specific QOL, and mental health outcomes. We hypothesized that both dietary and PA interventions would lead to improvements in generic and CRC-specific QoL and levels of anxiety and depression in comparison to a usual care group with a history of diagnosis of and treatment for CRC.

## Methods

The “Moving Bright, Eating Smart” study was a 2 × 2 factorial design RCT. Details of the trial protocol and the findings of the dietary and PA outcomes have been published elsewhere [14, 15]. This paper reports on the effects of interventions on generic and CRC-specific QoL, levels of anxiety, and depression.

Ethics approval was obtained from the Institutional Review Board of the Hong Kong West Cluster, the Hospital Authority in Hong Kong (UW12-478), and site-specific approval provided by other participating centers (Island East HKEC-2012-068; and Kowloon West KW/EX-13-002(59-02). Written informed consent was obtained from all participants, and the RCT has been registered with ClinicalTrials.gov (NCT01708824).

### Eligibility criteria and subject recruitment

Adults (aged 18 years or older) with histologically confirmed CRC and within 1 year of completion of primary cancer treatment were recruited from the surgical and oncological departments of four public hospitals in Hong Kong. CRC survivors with persistent or recurrent disease at the time of recruitment, with hereditary CRC syndromes, and with known contraindication to PA, such as wheelchair-bounded or chronic heart failure, were not eligible to participate. Potential eligible patients were invited to complete a validated food frequency questionnaire [16] and the Global Physical Activity Questionnaire [17]. Those who were already meeting the target of dietary intake (consumed less than five servings per week of red/processed meat and less than two servings daily of refined grains) or PA targets (accumulated more than 300 min per week of moderate-to-vigorous intensity PA) were excluded.

The study recruited 224 CRC survivors. Sample size calculation was based on the assumption that 25% of intervention group participants and 10% of the control group participants would meet the intervention targets with a power of 80% and a significance level of 5% and assuming a 10% dropout rate.

### Randomization and blinding

Eligible participants were randomized to either Group A (dietary and PA interventions, *n* = 55), Group B (diet only, *n* = 56), Group C (PA only, n = 56), or Group D (usual care without intervention, *n* = 56) using a block randomization method stratified by stoma status and study center, by a staff not involved in the study. Group allocation concealment was ensured by having a staff not involved in the study to keep the randomization schedule. The staff was phoned for group allocation when a patient was recruited. Due to the nature of the intervention, blinding of the participants and interveners were not possible. However, staff who assessed the outcomes were blinded.

### Interventions

**Fig. 1:**
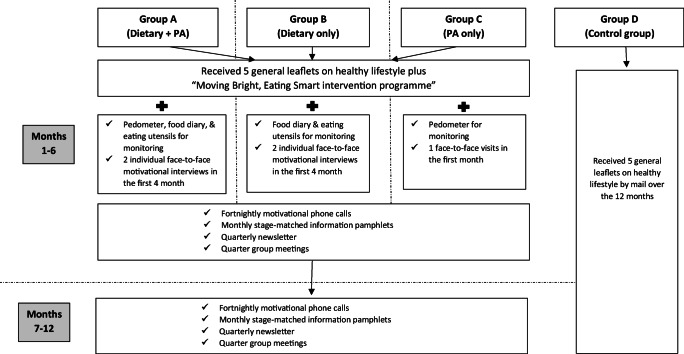
The intervention schedule of the “Moving Bright, Eating Smart” trial

The intervention adopted a personalized and multiple-contact approach and was based on the theory of planned behavior (TPB) [18] and the health action process approach (HAPA) [19]. The intervention details have been published elsewhere [15]. In brief, the content and the pace of the intervention delivered depended on the individual participant’s HAPA stage , namely pre-intentional, intentional, and action stage, respectively. Specifically, three sets of HAPA stage-of-change matched information leaflets for each of the dietary and PA interventions were developed to address the needs of pre-intenders, intenders, and actors. Participants of Group A (dietary and PA interventions), Group B (diet only), and Group C (PA only) received the intervention for 12 months and were followed for another 12 months. Figure 1 shows the intervention schedule.

#### Dietary interventions

The dietary interventions aimed to reduce red/processed meat consumption to less than five servings per week (less than two servings of processed meat) and to limit refined grains to two servings per day. A staggered approach based on the TPB and HAPA was adopted. Specifically, participants were encouraged to gradually reduce red/processed meat and to replace them with other protein sources, followed by replacing refined grains with whole grains. The intervention consisted of individual face-to-face motivational interviews (2 sessions, delivered by dietitians), motivational phone calls every 2 weeks by trained research assistants, monthly HAPA stage-of-change matched information pamphlets and quarterly newsletter by mail, and quarterly group meeting during the first 12 months. During the intervention period, participants were encouraged to self-monitor their red/process meat, refined, and whole grains intakes by completing monthly dietary intake logs. A set of eating utensils, including bowl, cups, and spoons, was provided to participants to facilitate portion size estimation.

#### Physical activity interventions

Participants were encouraged to increase PA progressively to achieve the general health target of 30 min of moderate-to-vigorous PA (MVPA) 5 days a week in the first 6 months and progressing toward the target of 60 min of MVPA 5 days a week over the next 6 months. Similarly, participants received individual face-to-face motivational interviews (1 session, delivered by a trained RA), motivational phone calls every 2 weeks, monthly HAPA-stage-of-change matched information pamphlets, a quarterly newsletter for experience sharing among participants by mail, and quarterly group meeting over the first 12 months. All participants were given a pedometer and a monthly PA log for tracking their levels of PA.

#### Usual care

Participants in the usual care (control) group received five pamphlets containing general healthy lifestyle advice, including healthy eating, increase PA, maintaining a normal body weight, quit smoking, and avoid alcohol abuse. The pamphlets were posted to participants at every 2 months during the initial 12 months.

### Measurements

Participants were assessed at baseline, 6, 12, 18, and 24 months. Generic health-related QoL was assessed using a Chinese version 12-Item Short Form Health Survey (SF-12) [20], and the CRC-specific QoL was assessed by the Functional Assessment of Cancer Therapy-Colorectal (FACT-C) scale [21], while anxiety and depression scores were obtained using a Chinese version of the Hospital Anxiety and Depression Scale (HAD-C) [22, 23].

### Generic quality of life

The SF-12 is widely used for assessing health-related QoL. The validity and reliability of SF-12 has been confirmed among ethnic Chinese [20, 24]. It consists of 12 items which are categorized into the subscales of Physical Component Score (PCS) and Mental Component Score (MCS). The SF-12 PCS and MCS scores were calculated using standard algorithms with a higher score implying better QoL. In addition, a population-specific algorithm was employed to convert the completed SF-12 into a single index, the six-dimensional health state short form (SF-6D) [25]. The SF-6D utility index scores ranged from 0.0 (representing the lowest state of QoL) to 1.0 (the highest state of QoL). SF-6D utility index is a preference-based health-related QoL measure that could be used for economic evaluation [25].

### CRC-specific quality of life

The FACT-C is a valid and reliable measure for assessing health-related QoL in CRC patients [21]. It consists of 27 core items evaluating physical well-being, social/family well-being, emotional well-being, and functional well-being plus nine additional colorectal cancer-specific items. Each item was rated on a 5-point Likert scale (0 to 4). The total scores (FACT-C, range from 0 to 132) and a general score (FACT-G, excluding the colorectal cancer-specific items, range from 0 to 104) were calculated. A higher score indicates better QoL. The FACT-C and FACT-G have been extensively tested in CRC survivors (including Chinese populations) and found to be reliable and valid measures [21].

### Anxiety and depression

The Hospital Anxiety and Depression Scale (HADS) was used to determine the levels of anxiety and depression [22, 23]. The HADS consists of 14 items and two subscales (anxiety and depression) with seven items in each subscale. Each item is scored on a 4-point Likert scale (0 to 3). Total scores were calculated for both subscales by summing up the individual items. Higher scores represent higher levels of depression and anxiety. The Chinese version of the HADS has been validated for the Chinese population [23].

### Statistical analysis

The intervention effects were analyzed by the intention-to-treat principle. To test the hypotheses of a difference between dietary intervention and usual care, and that between PA intervention and usual care, we used the linear mixed model that accounts for the extra-covariance among the repeated measurements over time. These two by two-group comparisons were made under the 2 (diet vs usual care) × 2 (PA vs usual care) factorial design that allows simultaneous assessment of both the dietary and PA interventions effects over usual care at a smaller sample size provided there is no interaction effect between the two interventions. Hence, we first examined the interaction effect between the dietary and PA interventions, before assessing their marginal effects.

Specifically, we first conducted a linear mixed model of SF-12 PCS, with random intercept and fixed effects of diet (vs usual care), PA (vs usual care), time, diet by PA interaction, diet by time interaction, PA by time interaction, the baseline value, stoma status, and study center. We did not observe a significant diet by PA interaction effect, which assured the appropriateness of analyzing the dietary and PA intervention effects under the 2 × 2 factorial design setting. After removing this and other insignificant interaction effects, we repeated the linear mixed model, based on which the coefficients and 95% confidence intervals (CI) corresponding to diet and PA were reported. The same analysis was conducted for other generic health-related QoL scores (SF-12 MCS, SF-6D utility index), the CRC-specific QoL scores (FACT-G and FACT-C scores), and levels of anxiety and depression. All statistical analyses were conducted using SAS version 9.4 (SAS Institute Inc., Cary, North Caroline), and a 5% significance level was used.

## Results

### Participants

A flow diagram of participants through the study has been reported previously [14]. In brief, 223 eligible participants (82 females, 141 males) were randomized in a 1:1:1:1 ratio to Group A, B, C, and D. Table 1 shows the baseline characteristics of participants. The mean age of the participants was 65.2 years (standard deviation = 10.1; range = 25 to 86). Most of them (87%) had completed at least college. Overall, 60% of the participants were colon cancer survivors (*n* = 133), 40% of them were rectal cancer survivors (*n* = 89), and one with synchronous colon and rectal cancers. There were 20% participants with stage I cancer, 43% with stage II, and 37% with stage III or IV. All participants had surgeries, and 30 of the 223 participants (13%) had a permanent or temporary stoma. About 60% participants (*n* = 129) received adjuvant chemotherapy and 19% (*n* = 43) received adjuvant radiotherapy. No significant differences in baseline characteristics among the four groups were observed.

**Table 1:** Baseline characteristic of participants

	Group A^1^ (Dietary + PA) (*n* = 55)		Group B^1^ (Dietary only) (*n* = 56)		Group C^1^ (PA only) (*n* = 56)		Group D^1^ (Usual care control) (*n* = 56)		*P* value
Age, years (mean, SD)	63.2	(11.4)	65.9	(9.8)	66.6	(9.5)	64.9	(9.4)	0.324
BMI, kg/m^2^ (mean, SD)	23.8	(3.3)	24	(3.2)	23.8	(3.1)	23.9	(3.6)	0.987
Sex									0.221
Male	37	(67)	34	(61)	40	(71)	30	(54)	
Female	18	(33)	22	(39)	16	(29)	26	(46)	
Education level									0.238
High school or below	7	(13)	6	(11)	11	(20)	4	(7)	
College or above	48	(87)	50	(89)	45	(80)	52	(93)	
Tumor stage^2^									0.407
Stage I	14	(26)	9	(16)	8	(15)	12	(21)	
Stage II	24	(44)	20	(36)	27	(49)	24	(43)	
Stage III or IV	16	(30)	27	(48)	20	(36)	20	(36)	
Stoma status									0.922
No stoma	47	(86)	48	(86)	48	(86)	50	(89)	
Permanent or temporary stoma	8	(14)	8	(14)	8	(14)	6	(10.7)	

^1^Values are number of participants (%) unless otherwise indicated

^2^Tumor stage were defined by the American Joint Committee on Cancer

*PA* physical activity

*n* number

*SD* standard deviation

*BMI* body mass index

At 24 months, 31 participants (14%) dropped out due to cancer recurrence (*n* = 18), loss to follow-up (*n* = 8), development of new cancer (*n* = 5), and died from cancer recurrence (*n* = 2). There were no significant differences in retention rates among all four groups and no significant differences in baseline characteristics between the completers and non-completers.

### Intervention adherence

Overall, participants randomized to the intervention groups (Group A, B, and C) attended an average of 95% of motivational sessions, answered 72% of the motivational phone calls and joined 44% of the group meetings. The attendance rates were similar among the three intervention groups. At 12 months, 55% and 43% of the participants receiving the dietary intervention (Group A and B) met the red/processed meat and refined grain targets, respectively. Of the participants receiving the PA intervention (Group A and C), 62% met the general health target (30 min of MVPA 5 days), and 55% met the cancer outcome target (60 min of MVPA 5 days).These positive behavioral changes were sustained at 24-month follow-up with 52%, 41%, and 60% of participants meeting the red/processed meat, refined grains, and cancer outcome PA targets, respectively.

### Impact of interventions on generic and CRC-specific quality of life

Table 2 shows the health-related QoL scores from baseline to 24-month follow up. As no interaction effect was observed between the dietary and PA interventions, Table 3 summarized the confirmatory results of the dietary and PA interventions. Participants receiving dietary interventions showed a significant improvement in the SF-6D utility index scores (mean difference 0.031, 95% CI 0.002 to 0.059). At 12 months, participants receiving dietary interventions showed a greater improvement in the SF-6D utility index scores (mean difference 0.042, 95%CI 0.003 to 0.081, Table 3) and the FACT-G total score (mean difference 3.09, 95% CI 0.13 to 6.04) when compared to those who did not receive the dietary intervention. At 24-month follow-up, participants receiving the dietary intervention showed a greater improvement in both the SF-12 PCS scores (mean difference 2.57, 95%CI 0.69 to 4.45) and the FACT-G total scores (mean difference 3.14, 95%CI 0.23 to 6.04) compared to those who did not received dietary interventions.

**Table 2:** Mean and standard deviations of the health-related quality of life and mental health outcomes from baseline to 24 months

	Group A (Dietary + PA)		Group B (Dietary only)		Group C (PA only)		Group D (Usual care control)	
Outcome/month	Mean	(SD)	Mean	(SD)	Mean	(SD)	Mean	(SD)
SF-6D utility index (0–1)								
Baseline	0.80	(0.16)	0.76	(0.16)	0.79	(0.18)	0.78	(0.16)
Month 6	0.91	(0.12)	0.86	(0.16)	0.91	(0.12)	0.85	(0.16)
Month 12	0.90	(0.11)	0.89	(0.13)	0.85	(0.16)	0.86	(0.15)
Month 18	0.90	(0.13)	0.88	(0.15)	0.87	(0.15)	0.85	(0.15)
Month 24	0.92	(0.12)	0.89	(0.15)	0.86	(0.16)	0.90	(0.13)
SF-12 PCS (0–100)								
Baseline	46.7	(7.1)	45.7	(8.2)	46.0	(8.5)	46.4	(6.7)
Month 6	50.7	(5.4)	47.8	(8.6)	52.1	(4.6)	47.8	(7.2)
Month 12	50.1	(5.1)	49.2	(6.9)	48.3	(7.3)	47.8	(6.8)
Month 18	50.1	(6.2)	48.7	(8.5)	49.9	(7.6)	48.0	(7.8)
Month 24	51.5	(5.1)	49.6	(8.2)	47.9	(8.2)	48.4	(7.5)
SF-12 MCS (0–100)								
Baseline	53.2	(8.5)	50.1	(9.1)	53.1	(9.3)	50.2	(10.6)
Month 6	57.8	(6.4)	56.8	(7.7)	57.4	(7.4)	56.0	(8.8)
Month 12	59.5	(4.5)	57.8	(7.4)	56.7	(7.3)	58.0	(7.3)
Month 18	58.6	(6.7)	58.1	(6.9)	56.5	(9.4)	57.1	(8.6)
Month 24	59.9	(4.3)	58.7	(6.8)	57.7	(7.9)	60.1	(5.5)
FACT-C total score (0–132)								
Baseline	110.4	(18.2)	110.8	(16.1)	110.8	(16.1)	106.9	(16.6)
Month 6	122.7	(14.2)	122.7	(15.5)	122.9	(15.7)	120.6	(16.8)
Month 12	126.6	(9.6)	124.3	(12.6)	122.8	(14.8)	120.3	(15.5)
Month 18	123.7	(13.5)	124.2	(14.4)	124.6	(14.7)	121.3	(17.3)
Month 24	127.9	(11.0)	125.7	(14.8)	123.8	(15.7)	125.1	(14.6)
FACT-G total score (0–104)								
Baseline	82.8	(13.5)	83.0	(12.7)	83.0	(12.7)	79.1	(13.8)
Month 6	91.7	(11.0)	91.7	(12.5)	92.3	(12.5)	90.0	(13.3)
Month 12	95.8	(7.6)	93.2	(10.7)	92.0	(11.7)	90.2	(12.3)
Month 18	92.5	(10.5)	94.0	(10.7)	93.2	(11.9)	90.9	(13.8)
Month 24	96.4	(8.3)	95.2	(10.8)	92.5	(12.0)	93.9	(11.7)
HADS-anxiety (0–21)								
Baseline	10.6	(4.0)	11.1	(3.9)	10.1	(3.5)	10.7	(3.8)
Month 6	8.9	(2.4)	8.7	(2.4)	8.6	(3.0)	9.0	(3.2)
Month 12	8.4	(1.6)	8.3	(2.0)	8.5	(2.4)	9.1	(3.1)
Month 18	8.6	(2.2)	8.6	(2.7)	8.2	(2.2)	9.0	(3.3)
Month 24	8.0	(1.7)	8.6	(2.7)	9.1	(3.3)	8.4	(2.7)
HADS-depression (0–21)								
Baseline	11.9	(3.7)	11.4	(3.4)	12.0	(3.2)	11.8	(3.4)
Month 6	10.8	(3.4)	11.0	(3.1)	10.7	(3.1)	11.3	(3.2)
Month 12	9.3	(2.2)	9.6	(2.8)	10.9	(2.7)	10.7	(2.8)
Month 18	9.6	(2.6)	9.8	(2.6)	10.1	(2.8)	10.7	(3.6)
Month 24	8.5	(1.8)	10.1	(2.8)	10.4	(3.4)	10.1	(3.0)

*PA* physical activity

*SF-6D* the six-dimensional health state short form

*SF-12 PCS* the Physical Component Score of 12-Item Short Form Health Survey

*SF-12 MCS* the Mental Component Score of 12-Item Short Form Health Survey

*FACT-C* the Functional Assessment of Cancer Therapy-Colorectal Scale

*FACT-G* the Functional Assessment of Cancer Therapy-general score

*HADS* the Hospital Anxiety and Depression Scale

*SD* standard deviation

**Table 3:** Efficacy of dietary and physical activity interventions on health-related quality of life and mental health  outcomes at various time points

	Dietary interventions		PA interventions	
Outcome/month	Difference	95% CI	Difference	95% CI
SF-6D utility index				
Month 6	0.014	(− 0.023 to 0.052)	0.039	(0.002 to 0.077)
Month 12	0.042	(0.003 to 0.081)	− 0.018	(− 0.057 to 0.021)
Month 18	*0.036*	(− 0.001 to 0.074)	0.008	(− 0.030 to 0.045)
Month 24	*0.033*	(− 0.005 to 0.071)	− 0.011	(− 0.049 to 0.027)
SF-12 PCS				
Month 6	−0.44	(− 2.29 to 1.41)	2.85	(1.00 to 4.70)
Month 12	*1.64*	(− 0.27 to 3.55)	− 0.07	(− 1.99 to 1.84)
Month 18	0.50	(− 1.35 to 2.35)	1.21	(− 0.64 to 3.05)
Month 24	2.57	(0.69 to 4.45)	0.18	(− 1.69 to 2.06)
SF-12 MCS				
Month 6	0.49	(− 1.50 to 2.48)	0.54	(− 1.46 to 2.54)
Month 12	1.25	(− 0.81 to 3.30)	− 0.52	(− 2.59 to 1.55)
Month 18	1.64	(− 0.35 to 3.62)	− 0.94	(− 2.94 to 1.05)
Month 24	0.39	(− 1.63 to 2.41)	− 1.23	(− 3.26 to 0.80)
FACT-C total score				
Month 6	1.75	(− 1.87 to 5.38)	− 0.17	(− 3.79 to 3.45)
Month 12	*3.51*	(− 0.25 to 7.26)	0.73	(− 3.03 to 4.49)
Month 18	1.67	(− 1.96 to 5.3)	− 0.23	(− 3.86 to 3.39)
Month 24	*3.32*	(− 0.37 to 7.01)	− 0.68	(− 4.37 to 3.01)
FACT-G total score				
Month 6	1.09	(− 1.75 to 3.94)	− 0.13	(− 2.97 to 2.72)
Month 12	3.09	(0.13 to 6.04)	0.69	(− 2.27 to 3.65)
Month 18	1.44	(− 1.41 to 4.30)	− 0.92	(− 3.77 to 1.93)
Month 24	3.14	(0.23 to 6.04)	− 1.05	(− 3.95 to 1.85)
HADS-anxiety				
Month 6	−0.17	(− 0.84 to 0.50)	0.11	(− 0.56 to 0.78)
Month 12	−0.56	(− 1.26 to 0.13)	− 0.03	(− 0.72 to 0.67)
Month 18	−0.15	(− 0.82 to 0.52)	− 0.22	(− 0.89 to 0.45)
Month 24	*−0.62*	(− 1.30 to 0.07	0.03	(− 0.46 to 0.53)
HADS-depression				
Month 6	−0.12	(− 0.87 to 0.63)	− 0.43	(− 1.18 to 0.32)
Month 12	−1.30	(− 2.08 to − 0.52)	0.01	(− 0.77 to 0.80)
Month 18	−0.59	(− 1.34 to 0.16)	− 0.38	(− 1.13 to 0.37)
Month 24	−0.93	(− 1.69 to − 0.16)	− 0.50	(− 1.27 to 0.26)

*PA* physical activity

*SF 12 SF-6D* the six-dimensional health state short form

*SF-12 PCS* the Physical Component Score of 12-Item Short Form Health Survey

*SF-12 MCS* the Mental Component Score of 12-Item Short Form Health Survey

*FACT-C* the Functional Assessment of Cancer Therapy-Colorectal Scale

*FACT-G* the Functional Assessment of Cancer Therapy-general score

*HADS* the Hospital Anxiety and Depression Scale

There was no significant time effect of the PA intervention on the QoL scores among the participants receiving the PA intervention (Table 2) indicating PA intervention did not have a significant effect on the generic and CRC-specific QoL scores. However, participants receiving the PA intervention showed a greater improvement in SF-6D utility index (mean difference 0.039, 95%CI 0.002 to 0.077, Table 3) and SF-12 PCS scores (mean difference 2.85, 95% CI 1.00 to 4.70) when compared to those who did not receive the PA intervention at 6 months.

### Impact of interventions on levels of anxiety and depression

Overall, participants receiving the dietary intervention showed a significant reduction in the levels of depression (mean difference 0.71, 95%CI 1.28 to 0.14, Table 2) but no significant changes in the levels of anxiety. Participants receiving the dietary intervention also reported a significantly greater reduction of levels of depression at both 12 months and 24 months when compared to those who did not receive the dietary intervention (Table 3). However, PA interventions were not significantly associated with changes in the level of depression. Also, neither dietary nor PA interventions were significantly associated with changes in the level of anxiety.

## Discussion

In the light of the growing number of CRC survivors, an evaluation of generic and CRC-specific health-related QoL of survivors becomes increasingly important. The major finding of this trial is that participants receiving the dietary intervention reported a significant improvement in generic preference-based QoL index (SF-6D utility scores), physical functioning (SF-12 PCS), the cancer-specific QoL scores (FACT-G), and levels of depression compared to those who did not receive the dietary intervention.

Health-related quality of life is an important aspect of successful cancer survivorship [26]. Encouragement of healthy diet and adherence to recommended dietary guidelines have been one of the goals of CRC survivorship care. The role of diet has been assessed previously as part of multiple health behavior change interventions for CRC survivors [9–11]. However, there are no published studies that have been undertaken among CRC survivors to assess the impacts of diet-only intervention. The present study adds to our understanding of the role that dietary interventions play on the health-related QoL and levels of anxiety and depression in CRC survivors.

Our results suggest that a theory-based dietary intervention has the potential to improve both generic and CRC-specific health-related QoL and reduce depression in CRC survivors. Cancer is a traumatic event. Cancer survivors often face adaptation problems and fears of cancer recurrence and negative effects of cancer treatment. Although the mechanisms of the observed beneficial effects of dietary intervention are not clear, research suggests that health-related behavior change interventions may promote posttraumatic growth in CRC survivors and foster positive growth and adaptation which may alleviate distress [9]. In addition, emerging evidence shows that provision of reliable and good quality health information may help to relieve distress in cancer survivors [27]. In current study, intervention components included individual face-to-face sessions delivered by dietitians plus regular phone calls and stage-of-change matched information pamphlets developed by oncology healthcare teams, and these may have contributed to the positive outcomes observed. However, it is unclear why there was no intervention effect on the levels of anxiety. A possible reason for the lack of intervention effects on anxiety could be due to relatively lower anxiety scores (mean scores ranged from 10.1 to 11.1) at baseline compared to the depression scores (mean scores ranged from 11.4 to 12.0). It is worth to note that a total score of 8 to 10 represents a borderline abnormal level of depression and anxiety, while a total score of 11–21 represent an abnormal level of depression and anxiety [22]. Further studies are needed to confirm and explain this observation.

CRC is a leading cancer burden worldwide; hence, economic evaluation is becoming more important for evaluating the cost-effectiveness of clinical interventions so as to inform resource allocation. A number of measures are available for estimating the health utility scores. However, no study had reported the effects of dietary or PA intervention on the health utility scores in CRC survivors. The current study has included the novel health utility data, the SF-6D utility index score. The SF-6D is a widely used preference-based measures of health derived from the popular health-related QoL instrument, SF-12 [28]. This tool applied cost-utility analysis in enabling the generation of quality-adjusted life years to guide health economic decisions. A novel finding of the current study is that a significant intervention effect on the SF-6D scores was observed for participants receiving PA interventions at 6 months and for participants receiving dietary interventions at 12 months. These novel results suggest that PA and dietary interventions are cost-effective in the short to medium term. More work is needed to confirm this observation and identify the most cost-effective approach of supporting CRC survivors.

While the effects of PA interventions on the generic and cancer-specific QoL in CRC survivors have been studied previously, we extended the evidence by examining both the long-term effects (24-month follow-up) and the impact on health utility scores. Consistent with the findings reported by other research groups [9, 29, 30], no significant between-group differences were observed on the CRC-specific QoL at any time points. The physical functioning measure (SF-12 PCS) and the SF-6D utility scores at 6 months did improve in the PA group during the intervention period. The PA intervention did not result in a significant improvement in participants’ PA levels [14]. As most participants were sufficiently physically active at baseline [13], the PA intervention might have not been able to yield significant increases in PA levels and, thereby, in health-related QoL among the participants. Ceiling effects of PA intervention have also been reported by other research teams [31]. Another explanation for the lack of a significant intervention effect may be related to the low intensity of the PA intervention (predominantly walking) used in current study. Participants received one face-to-face intervention session plus phone calls every 2 weeks and monthly pamphlets, without supervised PA training. Further studies are warranted to explore the optimal length, intervention intensity, and mode of delivery (for example, supervised PA training) of PA interventions for improving exercise behavior and QoL in CRC survivors.

The current study has several limitations that should be noted. First, the QoL, anxiety, and depression were based on self-report; although these measures are widely used in epidemiological and clinical researches, the inherent biases related to self-reporting cannot be fully eliminated. Second, this study was conducted in Chinese CRC survivors, which may limit the generalizability of the findings to other populations. Further studies are needed to establish the efficacy of the dietary intervention in other ethnicities and with other cancer types. The study was also limited by the possible ceiling effect of PA intervention. Future studies should recruit cancer survivors with lower PA levels at baseline or encourage greater intensities of PA intervention if deemed appropriate to confirm the effect of PA on health-related QoL.

In summary, this study provides insight into the use of dietary interventions for improving generic health-related and CRC-specific QoL and reducing depression in CRC survivors. Future efforts should be directed toward promoting behavior changes in CRC survivors to prevent cancer recurrence as well as to promote higher levels of QoL and reduced distress levels. Further studies with even longer-term follow-up are needed to assess the impact of dietary and PA interventions on clinical endpoints, such as disease-free survival and mortality, as well as the cost-effectiveness.
